# Severe Recurrent Gastrointestinal Bleeding Leading to the Diagnosis of Hereditary Hemorrhagic Telangiectasia: Clinical Response to Systemic Bevacizumab in a Case Report and Narrative Review

**DOI:** 10.7759/cureus.117000

**Published:** 2026-09-27

**Authors:** Btissam Essâdi, Ayoub Bouziane, Ouiam Elmqaddam, Hajar Koulali, Ghizlane Kharrasse, Zahi Ismaili, Abdelkrim Zazour

**Affiliations:** 1 Department of Hepato-Gastroenterology, Mohammed VI University Hospital Center, Mohammed First University, Oujda, MAR; 2 LAPOD (Research Laboratory of Pathology and Digestive Oncology) Faculty of Medicine and Pharmacy, Mohammed First University, Oujda, MAR

**Keywords:** argon plasma coagulation, bevacizumab, gastrointestinal bleeding, gastrointestinal telangiectasia, hereditary hemorrhagic telangiectasia, rendu-osler-weber disease

## Abstract

Hereditary hemorrhagic telangiectasia (HHT), or Rendu-Osler-Weber disease, is an inherited vascular disorder that may cause severe gastrointestinal bleeding, particularly at an advanced age. The management of diffuse and recurrent gastrointestinal involvement may remain challenging despite repeated endoscopic treatments. We report the case of a 62-year-old woman with recurrent epistaxis since childhood, nasal and cutaneous telangiectases, and severe recurrent gastrointestinal bleeding related to multiple gastric, duodenal, jejunal, and more distal small-bowel telangiectases. A definite diagnosis of HHT was established based on the presence of three Curaçao criteria. During the two years preceding systemic therapy, the patient required 11 hospitalizations, repeated packed red blood cell transfusions, intravenous iron infusions, and 13 sessions of argon plasma coagulation. Intravenous bevacizumab was initiated at a dose of 5 mg/kg every 15 days for six induction cycles, followed by 5 mg/kg every four weeks as maintenance therapy. During one year of follow-up, the patient remained free of gastrointestinal bleeding for the first six months. The first recurrence occurred during the seventh month. Three separate hospitalizations for recurrent bleeding were observed during the year following bevacizumab initiation, compared with 11 during the preceding two years. The two most recent episodes were associated with less severe anemia than the most severe episodes before treatment. Qualitative endoscopic improvement, with an apparent reduction in the number and prominence of antral telangiectases, was also observed. No significant bevacizumab-related adverse events were identified. In this patient with severe HHT-related gastrointestinal involvement, systemic bevacizumab was associated with a clinically meaningful reduction in the overall bleeding burden. This case highlights the importance of considering HHT in patients with multiple or recurrent gastrointestinal telangiectases associated with epistaxis and mucocutaneous telangiectases.

## Introduction

Hereditary hemorrhagic telangiectasia (HHT), also known as Rendu-Osler-Weber disease, is an autosomal dominant inherited vascular disorder with a prevalence classically estimated at approximately one in 5,000 individuals, although recent genomic data suggest that it is substantially underdiagnosed [1,2]. It is characterized by mucocutaneous telangiectases, which are small dilated superficial blood vessels, and visceral arteriovenous malformations (AVMs), particularly involving the lungs, liver, and central nervous system. Spontaneous recurrent epistaxis is the most common clinical manifestation and often begins early in life [1].

Gastrointestinal involvement generally becomes clinically significant later in life, most commonly during the fifth and sixth decades. Clinically significant gastrointestinal bleeding occurs in approximately one-third of adults with HHT and is mainly caused by gastrointestinal telangiectases predominantly involving the stomach and small bowel [1,3]. It most commonly presents as iron deficiency anemia secondary to chronic occult blood loss, whereas overt bleeding in the form of melena or hematemesis is less frequent. Severe forms may require repeated intravenous iron infusions and packed red blood cell transfusions and represent a major therapeutic challenge [1,3].

International guidelines recommend limited and targeted use of argon plasma coagulation (APC), an endoscopic non-contact thermal coagulation technique, and suggest intravenous bevacizumab or other systemic antiangiogenic therapies in moderate-to-severe forms [1]. The international multicenter InHIBIT-Bleed study notably demonstrated that systemic bevacizumab could improve anemia and significantly reduce transfusion and intravenous iron requirements in patients with HHT-related bleeding [4]. However, published evidence regarding systemic bevacizumab in patients with severe, diffuse gastrointestinal involvement remains limited.

We report the case of a 62-year-old woman with severe recurrent gastrointestinal bleeding due to diffuse gastroduodenal and small-bowel telangiectases, leading to a late diagnosis of HHT. Despite repeated endoscopic treatment, transfusions, and intravenous iron therapy, bleeding remained recurrent before systemic bevacizumab was initiated, after which a clinically meaningful reduction in bleeding burden was observed.

## Case presentation

A 62-year-old woman was admitted to our department for recurrent gastrointestinal bleeding, mainly hematemesis and melena, occurring generally every one to two months and sometimes at intervals of only 15 days, requiring repeated hospitalizations, packed red blood cell transfusions, and intravenous iron infusions. She reported spontaneous recurrent epistaxis since childhood. Previous nasofibroscopy had confirmed multiple bilateral nasal mucosal telangiectases requiring several cauterization procedures.

Her medical history included chronic liver disease of undetermined etiology, discovered incidentally. Viral, autoimmune, and metabolic etiological investigations were negative, with no alcohol consumption. The liver disease was initially compensated (Child-Pugh A6 [5], model for end-stage liver disease (MELD) 6 [6]), with liver stiffness of 18.9 kPa on FibroScan and no evidence of portal hypertension on initial imaging. Liver biopsy had been considered but deferred because of recurrent gastrointestinal bleeding.

The family history revealed recurrent epistaxis since childhood in two brothers and two of her children. One brother, who also had cirrhosis of undocumented etiology, died at the age of 52 following massive gastrointestinal bleeding. However, none of the relatives had undergone formal evaluation for HHT.

At the initial hospitalization, the patient was pale but hemodynamically and respiratorily stable, and physical examination revealed cutaneous telangiectases involving the cheek, anterior chest wall, and lower limbs (Figure 1).

**Figure 1:**
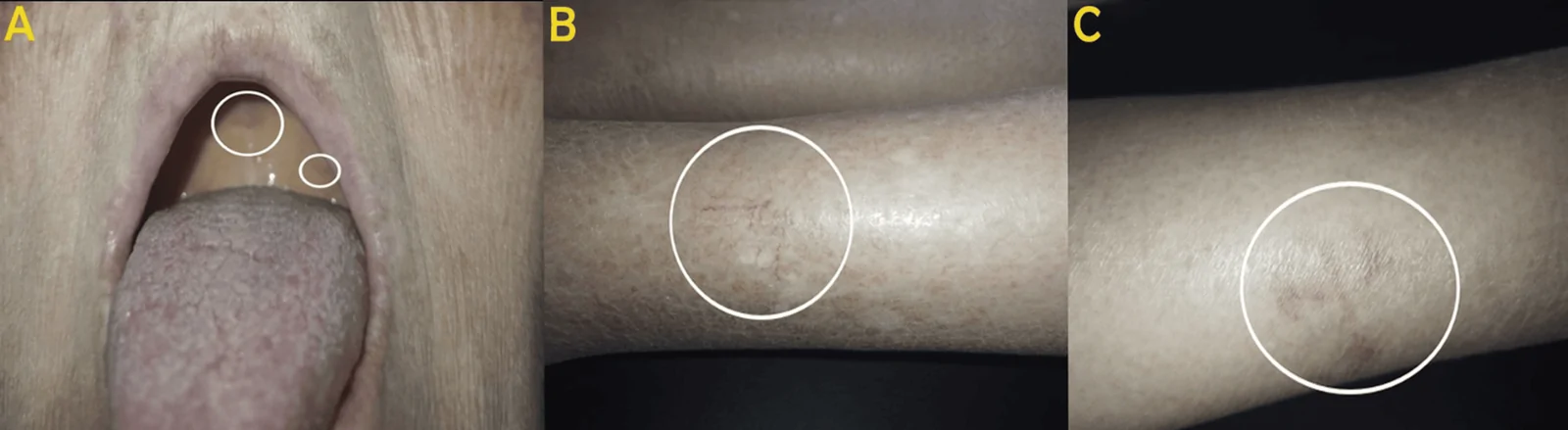
Characteristic mucocutaneous telangiectases in the patient with hereditary hemorrhagic telangiectasia (HHT): (A) telangiectasia of the lingual mucosa and (B, C) cutaneous telangiectases.

Laboratory investigations showed severe anemia with a hemoglobin level of 4.9 g/dL, mean corpuscular volume of 85 fL, mean corpuscular hemoglobin concentration of 31 g/dL, and serum ferritin of 14 ng/mL. Three units of packed red blood cells increased the hemoglobin level to 7 g/dL. The platelet count was 178,000/mm³, prothrombin time was 87%, INR was 1.0, and serum albumin was 36 g/L. Liver biochemistry showed no significant abnormalities except for an isolated elevation of gamma-glutamyl transferase to 70 U/L. The laboratory findings are summarized in Table 1.

**Table 1:** Laboratory findings at initial presentation.

Parameters	Patient Values	Reference Range
Hemoglobin	4.9 g/dL	12.0-16.0 g/dL
Mean corpuscular volume (MCV)	85 fL	80-98 fL
Mean corpuscular hemoglobin concentration (MCHC)	31 g/dL	32-36 g/dL
Serum ferritin	14 ng/mL	15-200 ng/mL
Platelet count	178,000/mm³	150,000-400,000/mm³
Prothrombin time	87%	70-100%
International normalized ratio (INR)	1.0	0.8-1.2
Serum albumin	36 g/L	35-50 g/L
Gamma-glutamyl transferase (GGT)	70 U/L	9-36 U/L

Upper gastrointestinal endoscopy revealed multiple antral, subcardial, and duodenal telangiectases without active bleeding, which were treated selectively with APC (Figure 2). Total colonoscopy with examination of approximately 15 cm of the terminal ileum revealed no colonic or ileal source of bleeding. Despite repeated APC sessions, bleeding recurred. A subsequent episode of massive gastrointestinal bleeding complicated by shock, with a hemoglobin level of 4 g/dL, required intensive care management and transfusion of four units of packed red blood cells. After stabilization, more extensive upper gastrointestinal examination using a colonoscope revealed multiple gastric, duodenal, and jejunal telangiectases, with the most clinically significant accessible lesions treated by APC.

**Figure 2:**
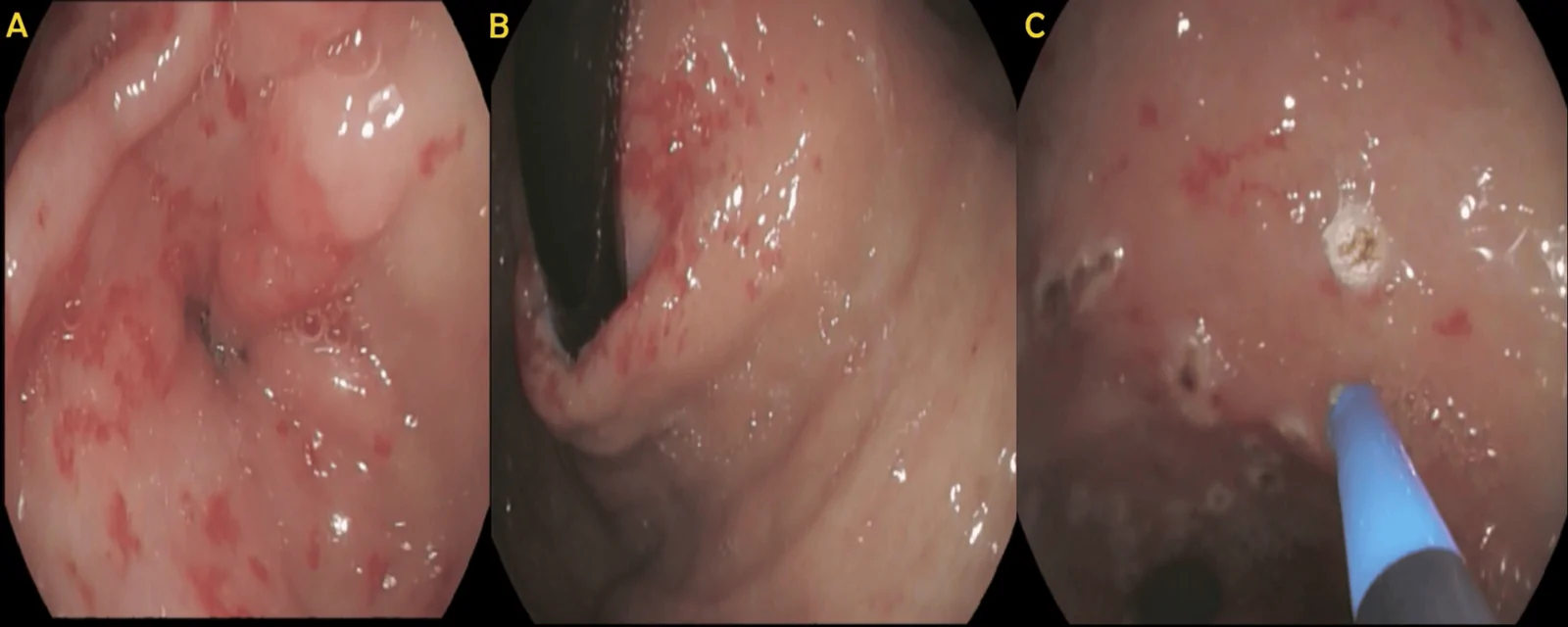
Gastric telangiectases and endoscopic treatment: (A) antral telangiectases; (B) subcardial telangiectases; and (C) targeted treatment with argon plasma coagulation.

Overall, during the two years preceding the introduction of systemic therapy, the patient required 11 hospitalizations for gastrointestinal bleeding, 13 APC sessions, repeated packed red blood cell transfusions, and intravenous iron infusions.

The presence of spontaneous recurrent epistaxis, characteristic mucocutaneous telangiectases involving the nasal and cutaneous sites, and visceral gastrointestinal involvement fulfilled three Curaçao criteria and therefore established a clinical diagnosis of definite HHT [7]. The family history was strongly suggestive but was not considered a formal criterion in the absence of a confirmed diagnosis in a first-degree relative. Genetic testing and screening of first-degree relatives were proposed but could not be completed. Cerebral magnetic resonance angiography, thoracoabdominopelvic CT angiography, and dedicated hepatic Doppler ultrasonography revealed no vascular malformation characteristic of HHT. Contrast echocardiography was not available at our center; therefore, conventional transthoracic echocardiography was performed and showed no significant abnormalities.

Because of persistent severe recurrent bleeding despite repeated endoscopic treatment, intravenous bevacizumab at a dose of 5 mg/kg every 15 days for six induction cycles, followed by 5 mg/kg every four weeks as maintenance therapy, was initiated. Monitoring of blood pressure, complete blood count, renal function, and proteinuria revealed no significant adverse events. Hemoglobin progressively increased to 9.7 g/dL, although anemia persisted. Following initiation of systemic bevacizumab, the patient reported complete resolution of epistaxis, with no further episodes documented during follow-up.

The patient experienced no gastrointestinal bleeding during the first six months following bevacizumab initiation. The first recurrence occurred during the seventh month, with hematemesis associated with melena. Endoscopy showed persistent gastroduodenal telangiectases treated with APC and, compared with the pretreatment examination, suggested a reduction in the number and prominence of antral telangiectases (Figure 3). This apparent endoscopic improvement was assessed qualitatively, as no validated endoscopic scoring system was used, and the potential contribution of concomitant APC cannot be excluded. Two small grade I esophageal varices were also present, without red signs or endoscopic stigmata of bleeding. No portal hypertensive gastropathy was observed.

**Figure 3:**
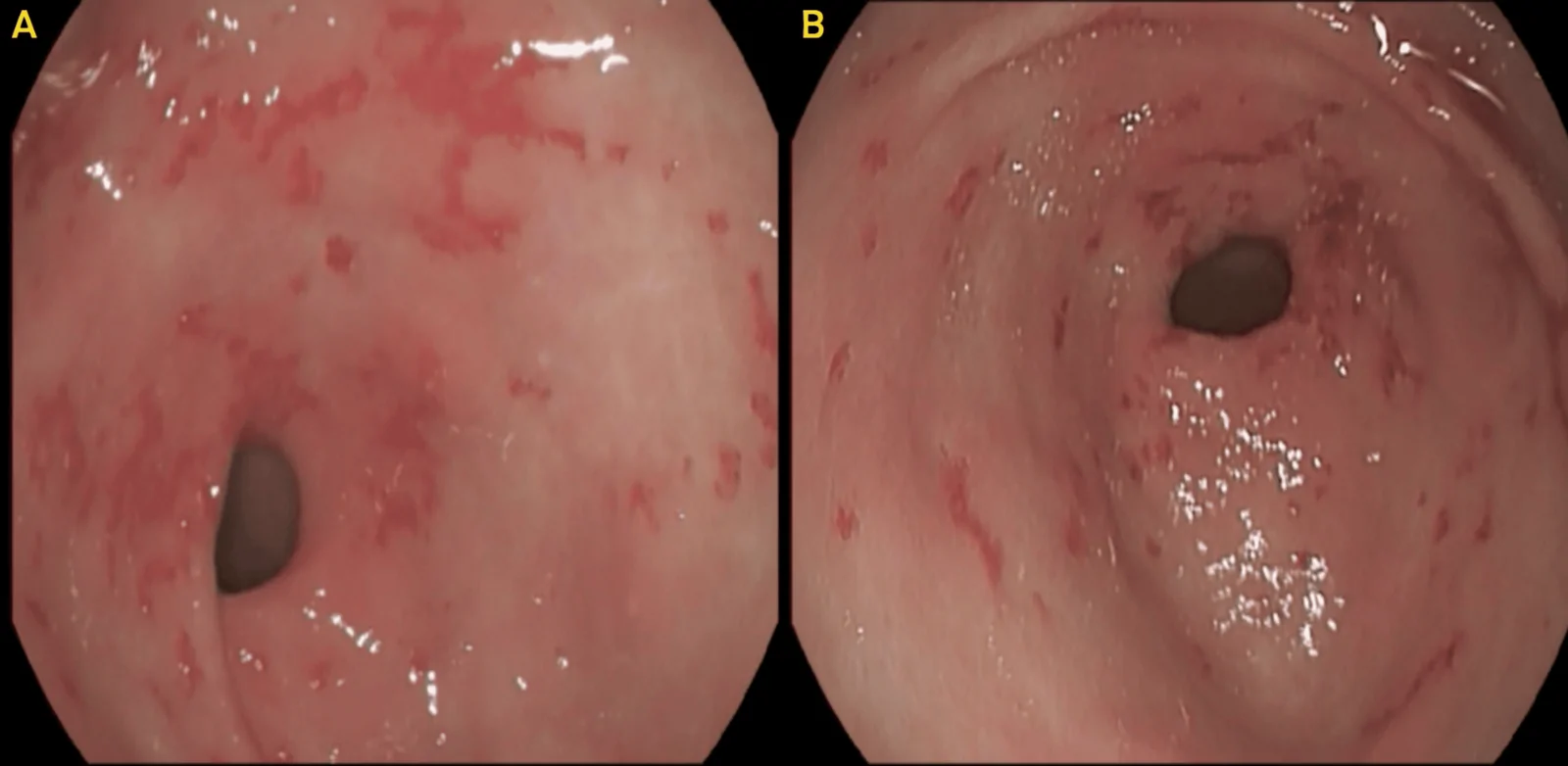
Endoscopic comparison before and after bevacizumab initiation: (A) upper gastrointestinal endoscopy before treatment and (B) upper gastrointestinal endoscopy at the seventh month after bevacizumab initiation, suggesting a reduction in the number and prominence of antral telangiectases.

Forty-eight hours later, during the same hospitalization, profuse hematochezia with hemodynamic instability occurred. CT angiography showed no active extravasation but now demonstrated ascites, splenomegaly, and portosystemic collaterals consistent with portal hypertension. Colonoscopy was negative, whereas video capsule endoscopy revealed multiple small-bowel telangiectases, several of which were actively bleeding. In the absence of endoscopic evidence of variceal bleeding or portal hypertensive gastropathy, these findings supported small-bowel telangiectases as the predominant bleeding source. Double-balloon enteroscopy was not available at our center, and these deep small-bowel lesions could therefore not be treated endoscopically.

Two additional bleeding recurrences occurred during the following five months, with respective hemoglobin levels of 7.5 and 8.2 g/dL. Both were managed with intravenous iron and targeted APC without the need for packed red blood cell transfusion. A scheduled bevacizumab infusion had been delayed during one of these active bleeding episodes and was administered one week after hospital discharge, without any causal relationship between this delay and the subsequent recurrence being established.

After one year of follow-up following bevacizumab initiation, three separate hospitalizations for recurrent bleeding had occurred, corresponding to three hospitalizations per patient-year, compared with 11 hospitalizations during the preceding two years, corresponding to 5.5 hospitalizations per patient-year. The post-treatment period also included an initial six-month interval without gastrointestinal bleeding. The two most recent recurrences, which were hematologically less severe than the most severe pretreatment episodes, did not require transfusion.

## Discussion

HHT is a multisystem autosomal dominant vascular disorder with a clinical prevalence estimated at approximately one in 5,000 individuals, although recent genomic data suggest substantial underdiagnosis [1,2]. Most molecularly confirmed forms are associated with pathogenic variants in ENG, which encodes endoglin, or activin A receptor-like type 1 (ACVRL1), and less commonly SMAD4 [8,9].

The pathophysiology of HHT is primarily based on disruption of the bone morphogenetic protein 9/bone morphogenetic protein 10 (BMP9/BMP10)-endoglin-ALK1-SMAD signaling pathway, which is essential for maintaining vascular endothelial homeostasis, quiescence, and maturation. This dysregulation promotes abnormal angiogenesis and excessive proangiogenic activity involving, in particular, vascular endothelial growth factor (VEGF), providing one of the pathophysiological rationales for the use of bevacizumab [8].

The clinical expression of the disease evolves with age. Epistaxis frequently begins during childhood or adolescence, whereas clinically significant gastrointestinal bleeding generally occurs after the age of 50 years [1,8]. Diagnostic delay therefore remains common. In the Comprehensive HHT Outcomes Registry of the United States (CHORUS), 63% of the first 600 participants were diagnosed only in adulthood, despite manifestations that had often begun much earlier [10].

Diagnosis remains primarily clinical and is based on the Curaçao criteria: spontaneous recurrent epistaxis, multiple telangiectases at characteristic sites, visceral involvement, and a first-degree relative with HHT. A definite diagnosis is established when at least three criteria are present [1,7]. Genetic testing is particularly useful in clinically equivocal cases and for family screening, as identification of a familial pathogenic variant allows targeted testing and appropriate surveillance of at-risk relatives [1,9].

Visceral AVMs may involve the lungs, brain, and liver and can cause severe complications including paradoxical emboli, brain abscesses, intracranial hemorrhage, high-output cardiac failure, portal hypertension, and ischemic biliary complications [1,8].

Gastrointestinal telangiectases predominantly involve the stomach and small bowel and cause clinically significant bleeding in approximately one-third of adults with HHT [1,3,10]. Chronic occult blood loss resulting in iron deficiency anemia is the most frequent presentation, whereas overt gastrointestinal bleeding is less common [1,3].

The severity of HHT-related gastrointestinal bleeding is primarily determined by its hematological impact and the level of supportive therapy required. International guidelines distinguish mild disease when the individualized hemoglobin target can be maintained with oral iron supplementation, moderate disease when intravenous iron is required, and severe disease when the hemoglobin target cannot be maintained despite adequate supplementation or when packed red blood cell transfusions are required [1].

Therapeutic management should be adapted to bleeding severity. Correction of iron deficiency is essential, with intravenous iron used when oral therapy is insufficient, whereas transfusions remain indicated in cases of severe symptomatic anemia or hemodynamic instability [1,3]. APC is the main endoscopic ablative technique for accessible gastrointestinal telangiectases, although international guidelines recommend limited and targeted use rather than systematic and repeated ablation of all lesions [1].

In a prospective series of 47 patients, Manfredi et al. reported a treatment response in 41 patients, with an increase in median hemoglobin from 7.0 to 11.9 g/dL and a reduction in transfusion requirements, although 27 patients required multiple endoscopic treatments [11].

Systemic therapy becomes particularly relevant in moderate-to-severe forms. Tranexamic acid may be considered in some mild forms of gastrointestinal bleeding, whereas systemic antiangiogenic therapy may be proposed for more severe disease [1,12]. Intravenous bevacizumab currently has one of the strongest bodies of evidence. In the multicenter InHIBIT-Bleed study involving 238 patients, bevacizumab resulted in a mean increase in hemoglobin of approximately 3.2 g/dL and a substantial reduction in transfusion and intravenous iron requirements [4]. Other systemic treatments, including thalidomide, pomalidomide, and pazopanib, have also been investigated, although data specifically concerning gastrointestinal bleeding are more limited [12,13].

Several features of our case deserve emphasis. First, diagnosis was particularly delayed despite recurrent epistaxis since childhood, documented nasal telangiectases, cutaneous telangiectases, and a strongly suggestive family history. HHT was ultimately recognized only at the age of 62 after several episodes of severe gastrointestinal bleeding. Three Curaçao criteria were clearly present, allowing a definite diagnosis of HHT despite the absence of molecular confirmation [1,7]. Recurrent childhood-onset epistaxis in several relatives also supports referral for genetic counseling and, if a pathogenic variant is identified, targeted family screening [1,9].

The gastrointestinal involvement was particularly severe, with diffuse telangiectases involving the stomach, duodenum, jejunum, and ileum. The patient experienced recurrent hematemesis and melena, including one episode complicated by hemorrhagic shock, with hemoglobin nadirs of 4-4.9 g/dL and repeated requirements for intravenous iron and packed red blood cell transfusions. This profile clearly meets current definitions of severe HHT-related gastrointestinal bleeding [1,3].

The therapeutic burden before systemic treatment was considerable, with 11 hospitalizations and 13 APC sessions over two years. APC had been used selectively on the accessible lesions considered most clinically significant. The need for repeated sessions should not be interpreted as an intrinsic failure of APC, but rather as a consequence of the diffuse, multifocal, and recurrent nature of the vascular lesions. Persistent severe recurrent bleeding despite repeated endoscopic treatments ultimately prompted the introduction of systemic antiangiogenic therapy.

Our patient received bevacizumab at a dose of 5 mg/kg every 15 days for six induction cycles, followed by 5 mg/kg every four weeks as maintenance therapy. This regimen is similar to those reported in the literature. VASCERN recommendations suggest an initial dose of approximately 5 mg/kg at intervals of 14 to 21 days, with subsequent treatment individualized according to response and tolerance [14]. Similarly, most patients included in the InHIBIT-Bleed study received four to six administrations of 5 mg/kg every two weeks, followed by continuous or intermittent maintenance therapy [4].

The clinical response was notable but incomplete. After two years marked by bleeding episodes occurring every one to two months and sometimes every 15 days, the patient experienced no gastrointestinal bleeding during the first six months following bevacizumab initiation. The first recurrence occurred during the seventh month.

Overall, three separate hospitalizations for recurrent bleeding occurred during the year following bevacizumab initiation, compared with 11 hospitalizations during the preceding two years. Although the pre- and post-treatment observation periods differed in duration, this evolution indicates a reduction in the frequency of events requiring hospitalization.

The biological evolution was also favorable during the initial phase, with an increase in hemoglobin to 9.7 g/dL. The two most recent recurrences were associated with hemoglobin levels of 7.5 and 8.2 g/dL, compared with nadirs of 4-4.9 g/dL before treatment. Both of the two most recent recurrences were managed with intravenous iron without packed red blood cell transfusion.

Qualitative endoscopic improvement was also observed. Compared with the pretreatment examination, upper gastrointestinal endoscopy performed during the seventh month after bevacizumab initiation suggested a reduction in the number and prominence of antral telangiectases. This finding should, however, be interpreted with caution because lesion burden was not assessed using a standardized endoscopic score, and exact comparability of visualization and anatomical extent between the pre- and post-treatment examinations could not be formally ensured. Concomitant APC sessions may also have contributed to the observed changes.

A narrative literature search was performed to identify previously published cases of HHT-related gastrointestinal bleeding treated with systemic bevacizumab. Several published cases have also described substantial reductions in bleeding and transfusion burden following systemic bevacizumab therapy despite considerable heterogeneity in baseline severity and treatment regimens [15-19]. The case reported by Bernardes et al. was particularly similar to ours: a 62-year-old woman with severe anemia, gastroduodenal telangiectases, and more than 100 small-bowel vascular lesions experienced prolonged clinical improvement after bevacizumab initiation [15]. Ou et al. reported a dramatic reduction in transfusion requirements in a patient with extremely severe diffuse gastrointestinal involvement [16], whereas Masood et al. observed a substantial reduction in hospitalizations and transfusion requirements following the induction phase [17]. Morrissey et al. also reported resolution of melena and transfusion requirements during treatment in a previously transfusion-dependent patient [18]. Finally, the case reported by Bertoli et al. illustrates the possible coexistence of chronic liver disease, portal hypertension, and HHT without the hepatic disease necessarily being directly related to HHT [19]. The main clinical characteristics, bevacizumab regimens, and reported outcomes of these cases, together with our case, are summarized in Table 2.

**Table 2:** Comparison of our case with five published cases of hereditary hemorrhagic telangiectasia (HHT)-related gastrointestinal bleeding treated with systemic bevacizumab.

Study	Patient	Presentation before bevacizumab	Bevacizumab regimen	Reported outcome
Our case	Female, 62 years	Diffuse gastric, duodenal, jejunal, and small-bowel telangiectases; hemoglobin (Hb) nadir 4 g/dL; hemorrhagic shock; 11 hospitalizations/2 years (5.5 per patient-year); 13 argon plasma coagulation (APC) sessions ; repeated transfusions and intravenous (IV) iron	5 mg/kg every 15 days × 6, then 5 mg/kg/month	No bleeding during the first 6 months; first recurrence during month 7; 3 hospitalizations during one year of follow-up (3 per patient-year); maximum Hb 9.7 g/dL; Hb during the last two recurrences 7.5 and 8.2 g/dL; qualitative endoscopic improvement; good tolerance
Bernardes et al. [15]	Female, 62 years	Melena; Hb 4.5 g/dL; gastric/duodenal lesions and >100 small-bowel lesions; repeated endoscopic treatments; high transfusion dependence	7.5 mg/kg every 3 weeks, then 5 mg/kg every 3 weeks	Rapid cessation of bleeding and transfusions; episode of melena after delayed infusion; response maintained at 1 year
Ou et al. [16]	Female, 58 years	Esophageal, gastric, duodenal, and jejunal lesions; repeated endoscopic treatments; extremely high transfusion requirements	5 mg/kg every 2 weeks	Major reduction in transfusion requirements; nearly 300 units of packed red blood cells estimated to have been avoided over 1 year
Masood et al. [17]	Female, 67 years	Gastric, duodenal, jejunal, and colonic lesions; 8 hospitalizations and 14 units of packed red blood cells in 1 year; repeated APC	5 mg/kg every 2 weeks × 6	Hb 9 → 13 g/dL; one hospitalization and one transfusion after induction
Morrissey et al. [18]	Male, 47 years	Diffuse gastrointestinal telangiectases; transfusion-dependent anemia; approximately 3-5 units of packed red blood cells/month; previous APC	5 mg/kg every 2 weeks × 6	Resolution of melena; substantial hematological improvement; no further transfusion during treatment
Bertoli et al. [19]	Male, 47 years	hereditary hemorrhagic telangiectasia (HHT) with severe epistaxis and melena; Hb as low as 2.8 g/dL; alcoholic cirrhosis with portal hypertension	2.5 mg/kg every 2 weeks for 24 weeks	Reduction in melena and epistaxis; increase in Hb; reduction in transfusions and IV iron without complete correction of anemia

These individual observations are consistent with the findings of the InHIBIT-Bleed study [4] and emphasize that clinical benefit does not necessarily imply complete cessation of bleeding. HHT remains a chronic vascular disorder, and bevacizumab does not permanently eliminate the underlying vascular substrate. Treatment response should therefore be assessed using several complementary parameters, including the frequency and severity of bleeding, hemoglobin level, iron and transfusion requirements, and number of hospitalizations [3,4].

The question of maintenance therapy remains particularly important. Some patients maintain a prolonged response after the induction phase, whereas others require regular maintenance therapy or additional courses when bleeding or anemia recurs [4,14]. Bernardes et al. reported an episode of melena following a one-week delay in bevacizumab administration [15].

In our patient, a scheduled infusion had also been delayed because of an active bleeding episode and was administered one week after hospital discharge. A subsequent recurrence was later observed. However, no causal relationship between the delay in bevacizumab administration and this recurrence can be established. This observation therefore does not allow any conclusion regarding an effect of treatment delay but highlights the ongoing uncertainties concerning optimal maintenance therapy schedules.

Treatment tolerance was favorable in our patient. Regular monitoring identified no hypertension, significant proteinuria, renal impairment, or other adverse events attributable to bevacizumab. Long-term monitoring nevertheless remains essential, as potential complications include hypertension, proteinuria, thromboembolic events, and impaired wound healing [4,14].

Chronic liver disease represents a distinct issue. Hepatic vascular malformations are a recognized manifestation of HHT and may include arteriovenous, arterioportal, or portovenous shunts, potentially resulting in high-output cardiac failure, portal hypertension, or ischemic biliary complications [1,20]. Hepatic Doppler ultrasonography is the first-line noninvasive examination for detecting and characterizing HHT-related hepatic vascular involvement, with multiphasic computed tomography or magnetic resonance imaging used as complementary modalities [20].

In our patient, chronic liver disease preceded the diagnosis of HHT. Elastography showed liver stiffness of 18.9 kPa, while the work-up for the main viral, autoimmune, and metabolic causes was negative, and there was no alcohol consumption. Initial imaging demonstrated morphological features of chronic liver disease without portal hypertension.

Dedicated hepatic Doppler ultrasonography, concordant with CT angiography, did not demonstrate hepatic vascular malformations characteristic of HHT. Three years later, the development of ascites, splenomegaly, portosystemic collaterals, and esophageal varices indicated the development of portal hypertension. However, no hepatic vascular malformation characteristic of HHT had been identified. A causal relationship between HHT and our patient's chronic liver disease therefore cannot be established.

The report by Bertoli et al. is informative in this regard. Their patient had HHT, severe gastrointestinal bleeding, cirrhosis, and portal hypertension, but the liver disease had been independently attributed to alcoholic steatohepatitis with fibrosis [19]. This observation illustrates the possibility of coexistence between chronic liver disease and HHT without the liver disease necessarily representing specific hepatic involvement of HHT.

Liver biopsy had initially been considered in our patient but was repeatedly deferred because of recurrent bleeding. After the diagnosis of HHT had been established, a noninvasive approach appeared particularly appropriate, as liver biopsy is generally discouraged when HHT-related hepatic vascular involvement is suspected because of the risk of bleeding [1,20].

Our case has several limitations. First, genetic testing and screening of first-degree relatives were proposed but could not be completed despite the strongly suggestive family history; nevertheless, the clinical diagnosis remains definite according to the Curaçao criteria. Second, the exact cumulative number of packed red blood cell units transfused and the total amount of intravenous iron administered before and after bevacizumab initiation are unavailable, preventing precise quantitative comparison of these parameters. The number of hospitalizations, APC sessions, hemoglobin evolution, and clinical course therefore represent the main objective criteria available.

Third, despite an extensive etiological work-up, the cause of the chronic liver disease remains undetermined. In addition, pulmonary screening included conventional transthoracic echocardiography rather than contrast echocardiography, although thoracic CT angiography did not demonstrate an identifiable pulmonary AVM. Finally, as with any isolated case report, the observational nature of the study and concomitant use of APC, intravenous iron, and transfusions when required do not allow the entire improvement to be attributed with certainty to bevacizumab alone. The endoscopic comparison was also qualitative and not based on a standardized lesion score.

Despite these limitations, the marked reduction in the frequency and severity of bleeding episodes after bevacizumab initiation, while endoscopic and hematological treatments had already been repeatedly used during the preceding two years without sustained control of recurrence, strongly suggests a clinical benefit of antiangiogenic therapy. This evolution is consistent with the findings of the InHIBIT-Bleed study and several published reports [4,15-19] and supports the use of systemic bevacizumab in selected patients with severe gastrointestinal involvement of HHT.

## Conclusions

HHT should be considered in patients with multiple or recurrent gastrointestinal telangiectases associated with epistaxis, mucocutaneous telangiectases, or a suggestive family history. In our patient with severe gastrointestinal involvement, systemic bevacizumab was associated with clinical benefit, including a reduction in the frequency and clinical impact of bleeding episodes. It may be considered in selected patients with severe HHT-related gastrointestinal bleeding. This case highlights the importance of early diagnosis, management tailored to disease severity, and appropriate family screening.
